# Emotions in trauma team

**DOI:** 10.1186/1757-7241-20-S2-P19

**Published:** 2012-04-16

**Authors:** Annette Jakobsen

**Affiliations:** 1 Faelles Akut Afdeling, Aarhus Universitetshospital, Denmark

## Background

This study shows how emotions are expressed in a trauma team setting and assesses their influence and importance with regard to team performance.

The term ”Team emotion” can be interpreted as the emotions that trauma team members do/do not display in a trauma receiving unit. Health care professionals are expected to refrain from showing emotion in the presence of patients and colleagues, but at the same time these emotions have a provable effect on teamwork.

## Methods

The study uses a qualitative, theme-based analysis of data acquired in semi-structured interviews with trauma team nurses in which they were asked to describe and assess their own emotions - and how these were expressed – during trauma receiving situations. Participant observation studies of the trauma team were also completed, along with audits and analysis of the medical records.

The study took place at a large Danish University Hospital, housing a Level One Trauma Centre.

## Results

The study confirmed the general belief that the showing of emotions, particularly if these are regarded as “negative” is not widely accepted within a trauma team. Displays of hope and/or happiness are acceptable, but emotions such as fear, anxiety, sadness and anger are considered inappropriate. Overt emotional response is considered more acceptable in certain professional groups than in others. From the interviews it was concluded that doctors were expected to be “above” emotional displays and the explicit norms within the organisation have a strong influence. In contrast, it is considered more acceptable for nurses to display emotion, although this should still be moderated by the norms and by the team.

## Conclusion

The study concludes that there is a tightly knit logical and empirical connection between team emotion and team communication. Strong emotions, such as pity towards patients and revulsion when faced with severe disfigurement must be moderated and used communicatively to assess the seriousness of a situation, but at the same time must not be repressed or ignored, despite the historical tendency to avoid or reject displays of emotion.

The study shows a need for more information and education, in relation to the effects of emotion on individuals and their work oriented interactions and relations.

